# Characterization of two dominant alleles of the major rhodopsin-encoding gene *ninaE* in *Drosophila*

**Published:** 2011-12-14

**Authors:** Amitavo Mitra, Yashodhan Chinchore, Ronald Kinser, Patrick J. Dolph

**Affiliations:** Department of Biological Sciences, Dartmouth College, Hanover, NH

## Abstract

**Purpose:**

In this study we investigated the biochemical and cell biologic characteristics of flies expressing two novel dominant alleles of the major rhodopsin encoding gene neither inactivation nor afterpotential E (*ninaE*) in a heterozygous background.

**Methods:**

Presence of the deep pseudopupil in flies was assayed 5 days post eclosion. For structural analysis, 1-μm-retinal cross sections were obtained from fixed and resin-embedded *Drosophila* heads. Confocal microscopy was performed on dissected retinas stained with antibodies specific for rhodopsin, NinaA, and F-actin. Rhodopsin levels were determined by western and slot blot analysis.

**Results:**

Dominant rhodopsin mutants showed progressive age-dependent and light-independent loss of the deep pseudopupil, without any apparent retinal degeneration at the morphological level. Expression of mutant rhodopsin caused rhodopsin to mislocalize to the cell body and the endoplasmic reticulum compartment. Mutant rhodopsin also caused loss of solubility of wild-type rhodopsin and its accumulation presumably as a high molecular mass complex in the photoreceptor cell body.

**Conclusions:**

In heterozygous mutant flies, there is loss of wild-type rhodopsin immunoreactivity on a western assay but less reduction using slot blot analysis. This suggests that mutant rhodopsin is likely inducing the misfolding and insolubility of wild-type rhodopsin. Localization of rhodopsin revealed that in mutant flies, wild-type rhodopsin is mislocalized to the cell body and the endoplasmic reticulum.

## Introduction

Retinitis pigmentosa (RP) is a diverse collection of genetically inherited diseases that is characterized by loss of visual acuity and retinal degeneration in humans [1-3]. The heterogeneity of the disease can be appreciated by the fact that RP can be inherited as an autosomal dominant (ADRP), autosomal recessive, or X-linked disease [4]. Despite the multimodal inheritance pattern, ADRP accounts for almost a quarter of all cases of RP [5,6]. Mutations in the rhodopsin gene account for the majority or most the underlying genetic determinants of ADRP cases in worldwide surveys [7-10], thus making the study of rhodopsin physiology in the context of RP an important avenue in elucidating the molecular mechanisms of RP. Despite the fact that mutations in a single gene (rhodopsin) are responsible for most cases of RP, mechanistic details might be complicated since in some cases not only does the same mutation in different people exhibit variability with respect to severity of their disease but also different amino acid substitutions at the same position can lead to distinct phenotypes [11,12].

The *Drosophila* phototransduction pathway has been studied in detail and has been established as a model system to elucidate mechanisms of retinal degeneration [13-15]. Even though the vertebrate and *Drosophila* phototransduction cascades have a different organization, they share anatomic and molecular features, making *Drosophila* an appropriate model. The *Drosophila* eye is a compound eye that consists of about 800 individual repeating units known as ommatidia. Individual ommatidia have about 20 cells out of which eight are photoreceptor cells. The phototransduction machinery in photoreceptor cells is localized to actin-rich microvillar structures known as rhabdomeres that are functionally equivalent to vertebrate outer segments. Loss of individual rhabdomeres within photoreceptors and/or the loss of the ommatidial array are indicative of retinal degeneration. The vertebrate and invertebrate light-stimulated signal transduction pathways are thematically similar, as evidenced by several common proteins [13,16].

Numerous rhodopsin mutations were isolated in *Drosophila* screens in the late 1960s [17-20], many of which cause retinal degeneration in fly photoreceptors. In a more recent screen, dominant neither inactivation nor afterpotential E (*ninaE*) alleles that undergo retinal degeneration have been isolated, some of which correspond to mutations in the same residues of human rhodopsin associated with ADRP [21]. Relevance for a *Drosophila* model of RP was further established when it was found that the most frequently occurring mutation in ADRP, a proline substitution at position 23 by histidine, faithfully recapitulated the dominant degenerative phenotype when engineered into the *Drosophila* rhodopsin gene [22].

Quantification of the rhodopsin present in such mutant flies, especially for rhodopsin mutants, is a widely used assay in all studies, but the lack of any detailed insight into the fate of rhodopsin has led to questioning how low levels of rhodopsin lead to rhodopsin-mediated retinal degeneration. The endoplasmic reticulum (ER) has been implicated to play a role in ADRP [10,23], and only recently has the importance of the accumulation of misfolded rhodopsin and its clearance mechanism been elucidated [24,25]. Accumulation of rhodopsin in photoreceptors, which potentially can be prone to aggregation and/or resistant to proper maturation/degradation, may contribute to the underlying mechanism(s) of retinal degeneration in phototransduction mutants, which otherwise show variability in functional and morphological phenotypes.

In this study we report two new alleles of the *Drosophila* major rhodopsin gene (*ninaE*), *ninaE^R11^* and *ninaE^R12^*. These two alleles are dominant and exert their effect in a light-independent manner. Using a combination of cell biologic and molecular approaches, we describe characteristics of these two novel alleles along with three previously characterized alleles as a comparison. We show that in heterozygous mutant flies, there is a significant accumulation of rhodopsin, part of which may be mislocalized to the cell body and the ER along with the disruption of the ER pattern within the cell body of photoreceptors. Mutant rhodopsin expression exerts its dominant effect by inducing misfolding and insolubility of wild-type rhodopsin.

## Methods

### *Drosophila* stocks

*Drosophila melanogaster* stocks *ninaE^5^*, *ninaE^D1^*, and *ninaE^D2^* were obtained from the Bloomington stock center (Bloomington, IN). The *ninaE^R11^* and *ninaE^R12^* alleles were identified in a previous arrestin 2 (*arr2*) degeneration enhancer screen [26]. All *ninaE* alleles were crossed into a *white* (*w^1118^*) background, and progeny were collected that were haploid for mutant and wild-type rhodopsin. Flies were reared in constant darkness.

### Deep pseudopupil analysis

At least 15 flies of each genotype were reared in complete darkness for 5 days and tested for the presence of deep pseudopupil (DPP) at 24-h intervals. Flies with a distinct DPP were given a score of 5; flies with faint to diffuse DPP were given a score of 3; and flies with no DPP were given a score of 0. The average of the observations was reported as the DPP score of the fly population within each genotype for each day.

### Histological fixation and sectioning

Heads of flies were bisected and immersed in ice-cold 2% glutaraldehyde in 0.1 M phosphate buffer. An equal volume of 2% osmium tetroxide was added and incubated for 30 min on ice. The glutaraldehyde/osmium tetroxide solution was removed, and the eyes were washed with 0.1 M phosphate buffer, followed by treatment with 2% osmium tetroxide for 60 min. The eyes were then serially dehydrated with increasing ice-cold ethanol (15%, 30%, 50%, 70%, and 90%) for 10 min each on ice. This was followed by two 10-min incubation periods in 100% ethanol at room temperature and two 10-min incubations in propylene oxide (Electron Microscopy Sciences, Hatfield, PA). The heads were incubated in a 50% propylene oxide/50% Durcupan ACM Fluka (Sigma, St. Louis, MO) mixture overnight on a rotator at room temperature, followed by a 4-h incubation in 100% Durcupan. Eyes were then embedded in 100% Durcupan and cured at 70 °C overnight. Cross sections (1 μm) were cut using a Sorvall ultra microtome MT-1 (Sorvall, Newtown, CT). The sections were stained with toluidine blue and observed on a Zeiss Axioplan 2 microscope using a 63×/2 numerical aperture (NA) oil immersion objective. Digital images were captured using an Optronics DEI-750 camera Optronics DEI-750 camera and associated software (Carl Zeiss Optronics, Oberkochen, Germany). At least three flies were analyzed for each genotype and treatment paradigm.

### Western and slot blot assay

Three fly heads of each genotype were homogenized in 25 μl Laemmli 1× sodium dodecyl sulfate (SDS) loading buffer. Each lysate (20 μl) was subjected to SDS PAGE (SDS–PAGE) followed by western analysis [26]. The primary antibodies used were anti-Rhodopsin1 (*Rh1*; 1:1,000; Developmental Studies Hybridoma Bank, Iowa City, IA), anti-Rh1–1D4 (1:100,000; gift from Dr. John Hwa, Weill Cornell Medical College, New York, NY), anti-Rab7 (1:10,000) [27], and anti-Arrestin2 (Arr2; 1:1,000). Secondary antibodies used were horseradish peroxidase (HRP) conjugated antimouse or antigoat (1:5,000). Arr2 and Rab7 were used as loading controls.

Densitometric analysis of Rh1, Arr2, and Rab7 was performed using the BioRad ImageQuant software (Amhersham Bioscience, Piscataway, NJ). Each experiment was repeated independently three times.

For slot blot analysis, two wild-type heads were ground in 20 μl 1× SDS loading buffer, and the total volume was adjusted to 200 μl with 1× SDS loading buffer/1 M urea in 0.5× PBS (0.15 M NaCl, 0.002 M KCl, 0.01 M Na_2_HPO_4_, 0.001 M KH_2_PO_4_, pH 7.4). Serial dilutions were prepared, and samples equivalent to 1, 0.5, 0.25, and 0.125 heads were applied to a BioDot apparatus (BioRad, Hercules, CA). OptiTran BA-S 85-supported nitrocellulose membrane (Whatman, Piscataway, NJ) was used for the protein transfer. For heterozygous mutants, lysate concentrations were doubled. The membrane was denatured by incubating in 0.2 M sodium hydroxide for 30 min at room temperature followed by blocking in 5% milk in 1× PBS+0.1% Tween-20 for another 30 min. Antibody incubations were performed as in western analysis.

Densitometric analysis of total Rh1 levels in wild-type and mutant flies was performed as described for western blot analysis. Total rhodopsin level in heterozygous mutant flies relative to wild-type flies was calculated and plotted by comparing rhodopsin levels of mutants at various concentrations to those of wild-type samples. Rhodopsin levels for each sample were arrived at by averaging the values obtained from three independent repetitions.

### Immunohistochemistry

Retinas from adult flies were prepared for whole-mount immunostaining as described previously [27]. The primary antibodies used were anti-Rh1 (1:50; Developmental Studies Hybridoma Bank, Iowa City, IA), anti-Rh1–1D4 (1–800; a gift from Dr. John Hwa, Yale University), anti-Rh1 (1:50; polyclonal antibody; gift from Dr. C. Zuker, Columbia University, New York, NY), and anti-NinaA (1:150; gift from Dr. Craig Montell, Johns Hopkins University, Baltimore, MD). Rhabdomeres were visualized by staining for F-actin using rhodamine or Alexa-568-conjugated phalloidin (Molecular Probes, Carlsbad, CA). Secondary antibodies were antimouse- or antirabbit-conjugated Alexa-488 or Alexa-647 (1:300; Molecular Probes).

Images were captured on a Leica TCS SP confocal laser-scanning microscope (Leica Microsystems, Heidelburg, Germany) and Nikon A1RSi Confocal Workstation (Nikon, Melville, NY). Image processing was done with Adobe Photoshop (San Jose, CA). Retinas from at least three flies in each genotype were processed.

### Statistical analyses

The Student *t* test was used for statistical comparison between wild-type controls and the various mutant genotypes. Differences were considered statistically significant at p<0.05.

## Results and discussion

### Progressive light-independent loss of the Deep Pseudopupil in *ninaE* heterozygous flies

**Figure 1:**
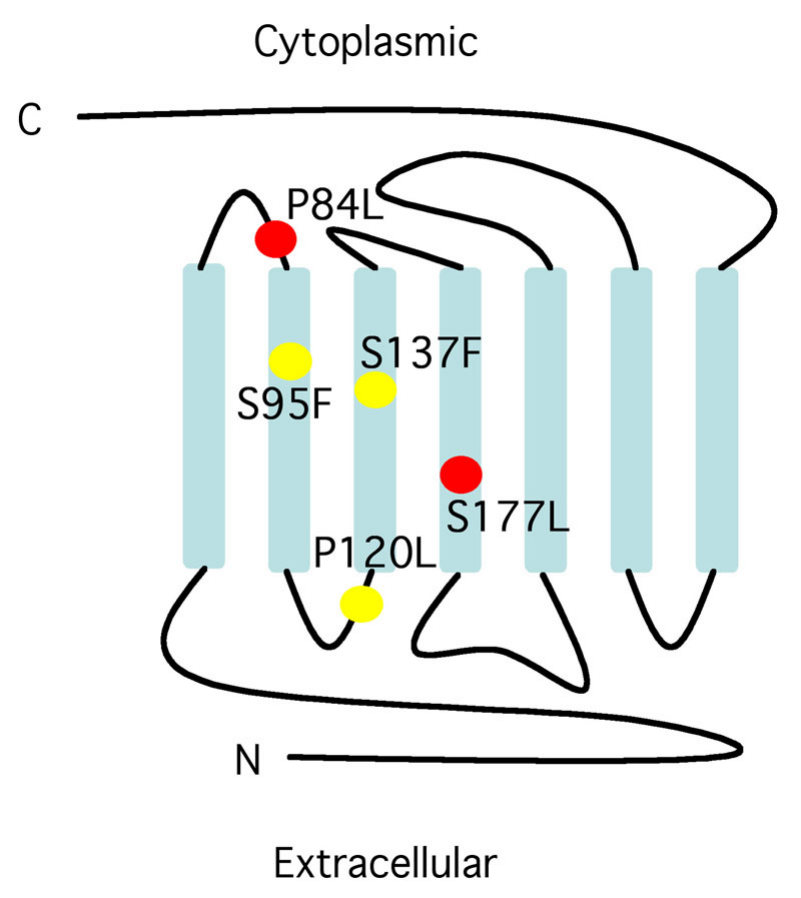
Schematic of rhodopsin protein and location of mutations. Yellow dots represent the position of previously published *ninaE* alleles used in this study. Red dots represent novel alleles of *ninaE*. The genetic change and corresponding allele names are as following: P84L, *ninaE^R11^*; S95F, *ninaE^D2^*; P120L, *ninaE^5^*; S137F, *ninaE^D1^*; S177L, *ninaE^R12^.*

Previously, we isolated a collection of genetic enhancers of *arr2* degeneration [26]. From that collection, two alleles were genetically mapped to the *ninaE* locus, which encodes for the major *Drosophila* rhodopsin, Rh1. Sequencing of the *ninaE* gene from these alleles indicated that they both had point mutations within the coding region. The *ninaE^R11^* allele has a proline to leucine mutation in the first cytoplasmic loop at position 84, and the *ninaE^R12^* allele has a serine to isoleucine mutation in the fourth transmembrane domain at position 177 (Figure 1).

**Figure 2:**
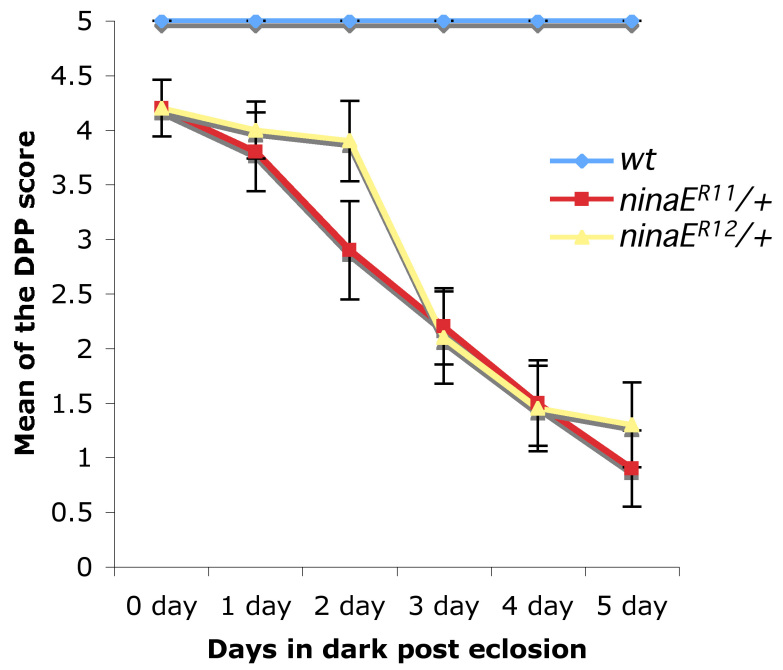
Heterozygous *ninaE* mutants show loss of deep pseudopupil. At least 15 newly eclosed *ninaE^R11/+^* and *ninaE^R12/+^* flies were reared in the dark for 5 days and deep pseudopupil (DPP) was assayed once per day. A graded scoring system was used wherein scores of 5, 3, or 0 were given to flies with intact, diffuse, or absent DPP, respectively. Note the age-dependent gradual loss of DPP in heterozygous mutant flies. The DPP loss in both *ninaE^R11/+^* and *ninaE^R12/+^* flies is statistically significant for all time points (p<0.05).

Since ADRP accounts for the majority of cases of diagnosed RP, we are primarily interested in the dominant phenotype exerted by the mutant protein on its wild-type counterpart. To test whether these rhodopsin alleles had dominant phenotypes, we reared heterozygous *ninaE* mutant flies in complete darkness and assayed them for loss of the DPP [28]. The DPP is a virtual image of the trapezoidal array of multiple rhabdomeres near the center of curvature of the *Drosophila* compound eye [29]. The loss of the DPP indicates a reduction in the amount of rhodopsin or a disruption in the ommatidial structure. Both *ninaE^R11/+^* and *ninaE^R12/+^* flies reared in the dark underwent progressive loss of DPP, with only about 10% of flies showing no loss of DPP by day 5 post eclosion (Figure 2). The loss of DPP in mutant heterozygous flies compared to control flies for every time point is significant with a p value less than 0.05. Although a small number of flies retained their DPP at the end of our assay time frame, the data clearly showed the trend of loss of the DPP in a light-independent manner. The continued presence of detectable DPP in a minor percentage of flies is likely due to the qualitative basis of the DPP assay and/or variation in the phenotype presentation within the fly population at the time of our assay. These data suggest that *ninaE^R11^* and *ninaE^R12^* are dominant alleles of *ninaE* and that the loss of DPP is age dependent and light independent, suggesting underlying retinal degeneration.

### Rhabdomeres of the mutants do not degenerate

**Figure 3:**
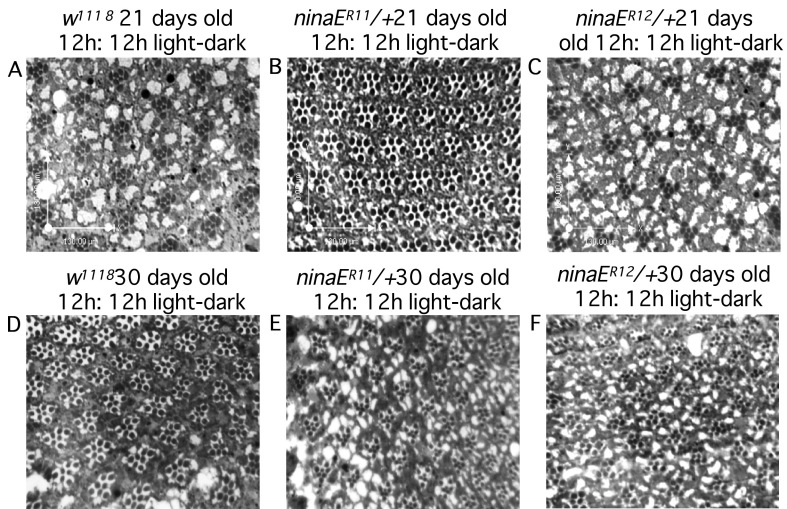
Heterozygous *ninaE* mutants maintain rhabdomeric integrity. Flies in panels (**A**-**C**) were processed 21 days post eclosion and reared in constant darkness at room temperature. Flies in panels (**D**-**F**) were processed 30 days post eclosion and reared in 12 h:12 h light–dark cycles. Note the distinct presence of rhabdomeres in the heterozygous mutant photoreceptors in both regimens.

Since heterozygous mutant flies show loss of DPP, we examined aged flies to observe the loss of rhabdomeres and/or loss of structural integrity of the ommatidial array. Flies aged for 3 weeks in complete darkness did not show any readily identifiable hallmarks of degeneration and had a full complement of seven rhabdomeres per ommatidium and a regular ommatidial array (Figure 3A-C). Moreover, analysis of heterozygous animals reared in a 12 h:12 h light–dark cycle for up to 30 days showed minimal changes in photoreceptor cell structure and rhabdomere morphology (Figure 3D-F). These results are also consistent with previous reports on published *ninaE* alleles. It should be noted that the previously identified *ninaE^D1^* dominant allele showed degeneration only when exposed to continuous light and showed minimal degeneration in dark-reared flies only after 40 days [30]. We also analyzed *ninaE^R11^* and *ninaE^R12^* homozygous flies under two different aging paradigms (7-day and 3-weeks old, dark reared) by means of cross sections and did not observe any noticeable retinal degeneration (data not shown).

The lack of major structural defects is not necessarily in conflict with the observed early loss of DPP data. It has been previously shown that the loss of DPP can be correlated to reduced rhodopsin content even when the photoreceptors have intact rhabdomeres [29,31]. Moreover, although rhodopsin is required for photoreceptor morphogenesis and viability [32,33], it has been previously shown that flies with a 70% reduction in photopigment level [34] or a reduction in rhodopsin levels to <3% in photoreceptors [35] have an overall normal photoreceptor morphology.

Three mutations in the human rhodopsin gene have been identified that cause congenital stationary night blindness [36-38]. In this form of retinopathy, similar to RP, loss of vision in low-light conditions is an early clinical observation, but unlike RP where there is a prominent loss of rods and cones, in congenital stationary night blindness there is no loss of rods and cones. These histological results are similar to those observed in the *Drosophila* mutants in this study as no loss of rhabdomeres in photoreceptor cells was observed.

### Rhodopsin is mislocalized in dominant mutants

Our histological data indicate that in mutant photoreceptors low levels of rhodopsin may be properly trafficked to the rhabdomeres to maintain rhabdomeric integrity. To test whether rhabdomeric rhodopsin is present in these flies and whether there is any mislocalization of rhodopsin, we stained whole retinas with anti-Rh1 and the ER-specific marker anti-NinaA. The *ninaA* gene in the *Drosophila* genome encodes for an eye-specific homolog of cyclosporine A-binding protein [39], which functions as an Rh1-specific chaperone and is involved in its maturation and transport from the ER to the rhabdomeric membranes [40-42]. We wanted to ascertain if, in our mutants, rhodopsin is localized to the ER, indicating maturation/trafficking defect/delay.

**Figure 4:**
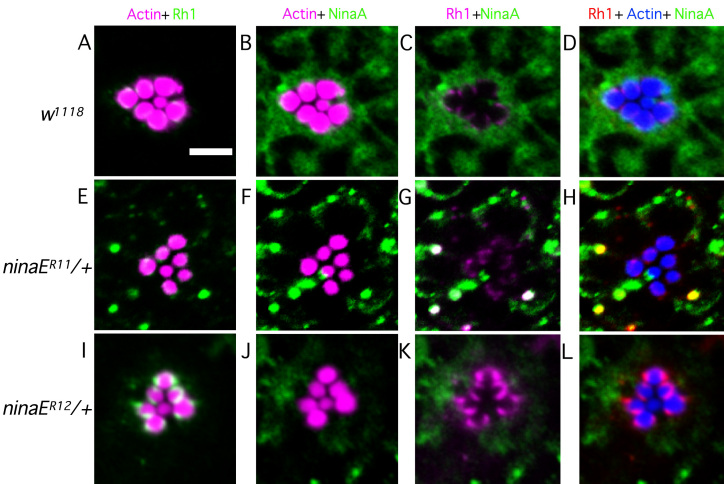
Rhodopsin and NinaA show improper localization in heterozygous *ninaE* mutants. Dark-reared fly retinas were isolated and triple stained for F-actin, Rhodopsin (Rh1), and neither inactivation nor afterpotential protein (NinaA). **A**-**D**: Wild-type photoreceptors show proper rhodopsin localization to the base of the rhabdomeres and NinaA is localized to the cell body. **E**-**H**: *ninaE^R11/+^* photoreceptors show partial proper rhodopsin localization with multiple rhodopsin- and ninaA-positive puncta present in the cell body. NinaA staining is restricted to the edges of the cell body. **I**-**L**: *ninaE^R12/+^* photoreceptors show rhodopsin localization to the base of the rhabdomeres, with diffuse rhodopsin staining in the cell body having few distinct rhodopsin-positive vesicles. NinaA stains diffusely throughout the cell body similar to the wild type. Scale bar represents 5 μm.

In dark-reared wild-type flies, Rh1 is localized to the base of rhabdomeres in a crescent-like shape and no cytoplasmic rhodopsin can be observed (Figure 4C). NinaA shows a diffused staining pattern throughout the cell body (Figure 4B). All mutant photoreceptors show some rhabdomeric localization of rhodopsin, suggesting that some proportion of rhodopsin may be correctly trafficked to its site of activity. This rhabdomeric rhodopsin may explain the long term viability of photoreceptors and lack of morphological defects. However, both mutants analyzed exhibit some defect in rhodopsin localization where a significant amount is mislocalized to the cell body.

In *ninaE^R11/+^* flies, a proportion of rhodopsin is mislocalized in a punctate-staining pattern in the cell body. Many of the large rhodopsin-positive puncta also stain positive for NinaA. In contrast to NinaA distribution in wild-type flies, NinaA in *ninaE^R11/+^* is limited to the cell body periphery and is no longer localized throughout the cell body (Figure 4E-H). Flies heterozygous for the *ninaE^R12^* mutation show extensive diffused rhodopsin mislocalization to the cell body that does not seem to localize with NinaA. In contrast, NinaA is localized in a wild-type distribution (Figure 4I-L). As we use NinaA as a marker for the ER, we speculate, based on the restrictive ER staining in *ninaE^R11^* heterozygous flies, that the normal ER distribution within photoreceptor cells in a mutant background may be affected. Another mutually nonexclusive possibility is that the majority of the NinaA is relocated within the ER to areas where rhodopsin is present and likely concentrated. We see similar results with the previously published dominant *ninaE* allele *ninaE^D2^* (data not shown). We also see normal NinaA distribution in another previously published dominant *ninaE* allele *ninaE^D1^* (data not shown); this distribution is similar to our observations in *ninaE^R12/+^* flies. This suggests that there exists heterogeneity within the dominant *ninaE* alleles in presentation of defects at the cellular level. This collection of data suggests that rhodopsin is mislocalized to the cell body and may also be accumulating in the ER, resulting in changes in the ER distribution pattern and/or localization of NinaA within the ER.

### Rhodopsin becomes insoluble in mutant photoreceptors

**Figure 5:**
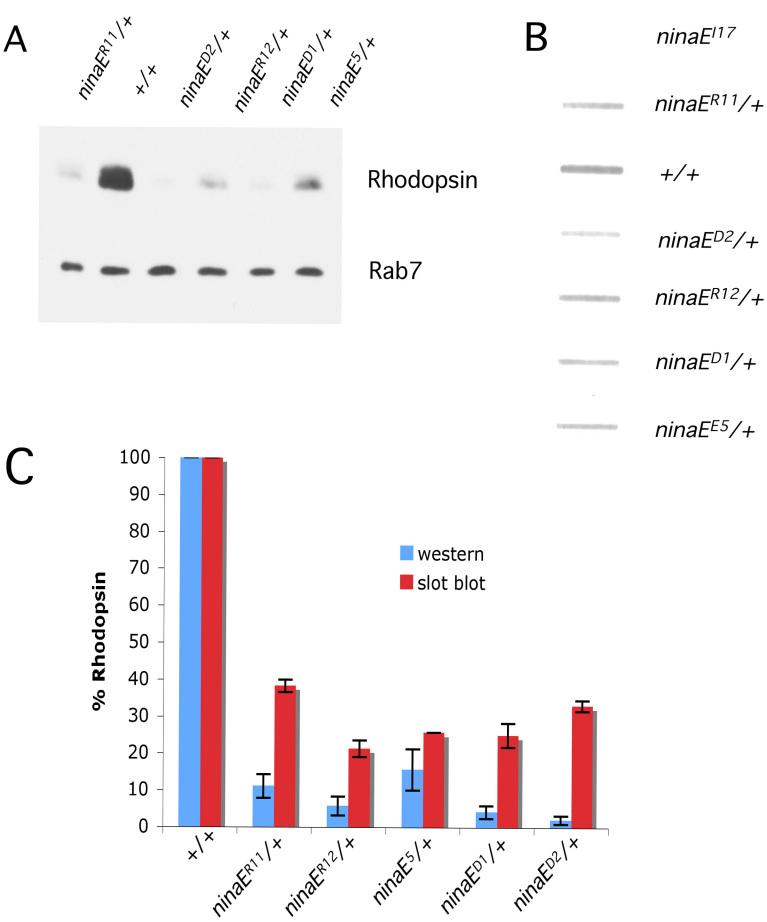
Quantification of soluble and total rhodopsin in heterozygous *ninaE* mutants. **A**: Lysates equivalent of four heads were loaded onto a sodium dodecyl sulfate PAGE (SDS–PAGE) gel and probed with anti-Rh1 antibody, while Rab7 antibody was used as a loading control for western analysis. **B**: Whole head lysates equivalent to two wild-type heads and four heterozygous mutant heads were prepared and diluted for slot blot analysis as detailed in the Methods and probed with anti-Rh1 antibody. **C**: The blots probed with anti-Rh1 antibody were quantified to reflect the relative level of soluble versus total rhodopsin in mutant flies. Note the loss of wild-type rhodopsin intensity in (**C**) when mutant rhodopsin is co-expressed in the same photoreceptors. The difference in the levels of soluble versus total rhodopsin in each of the genotypes tested is statistically significant (p<0.014). Error bars are represented as average % Rhodopsin±SD. Each column represents the average of three biologic replicates.

Previously it has been shown that mutations in rhodopsin cause a reduction in protein levels. When protein levels are determined by western blot analysis, *ninaE^R11/+^* and *ninaE^R12/+^* show significantly (10% [p<0.0001] and about 6% [p<0.001], respectively) rhodopsin levels relative to the wild type (Figure 5A,C). Since all of the animals analyzed were heterozygous and contained a haploid content of wild-type rhodopsin, the loss of rhodopsin immunoreactivity below 50% indicates that the mutant rhodopsin is triggering the loss of rhodopsin in this assay.

In human cases of ADRP, mutant rhodopsin has been shown to be prone to aggregation and to form high molecular weight complexes [43]. Abnormal accumulation of protein aggregates is also associated with diverse human pathologies, such as Huntington’s disease, Alzheimer disease, and spinocerebellar ataxia [44]. Therefore, we explored whether rhodopsin becomes insoluble in the dominant mutants. We reasoned that insoluble aggregated rhodopsin might not be detectable on immunoblots since the protein complexes may be unable to enter the polyacrylamide gel matrix. However, insoluble proteins may be detectable by slot blot analysis since this method does not require the proteins to be solublized. Interestingly, slot blot data indicate that there is significantly more rhodopsin in the dominant mutants than detected by western blot. Flies heterozygous for the *ninaE^R11^* and *ninaE^R12^* mutations show approximately twofold to fourfold more total rhodopsin than on western blots. These differences are statistically significant (p<0.001; Figure 5B,C). This is also evident in the immunolocalization data in Figure 4. Even though there is no detectible protein on a western blot, both *ninaE^R11^* and *ninaE^R12^* heterozygotes exhibit Rh1 immunoreactivity. In the case of *ninaE^R12^*, levels appear even higher than the wild type.

We were interested in whether other previously characterized rhodopsin alleles also show a difference between soluble (western blot) and total (slot blot) rhodopsin levels. We chose to analyze *ninaE^D1^*, *ninaE^D2^*, and *ninaE^5^* alleles (Figure 1). Similar to the *ninaE^11^* and *ninaE^12^* alleles in this study, these rhodopsin mutations have previously been shown to induce low levels of rhodopsin on western blots and minimal retinal degeneration [30]. *ninaE^D1/+^* and *ninaE^D2/+^* flies show a significant severe loss of rhodopsin immunoreactivity on a western blot, less than 10% of wild-type levels (p<0.0001; Figure 5A, C). This is comparable to previously described rhodopsin levels in these two mutants [30]. We have found that *ninaE^5/+^* flies exhibit approximately 15% rhodopsin levels (p<0.001) compared to about 1.5% in *ninaE^5/+^* flies relative to the wild type, as reported elsewhere [45] (Figure 5A,C).

However, using slot blot analysis, there is a significant sixfold (p<0.001) and 15-fold (p<0.05) more detectable rhodopsin in *ninaE^D1/+^* and *ninaE^D2/+^* flies, respectively, while *ninaE^5/+^* shows about threefold (p<0.05) more rhodopsin compared to wild-type levels (Figure 5B,C). These data suggest that rhodopsin in dominant *ninaE* alleles may not be efficiently degraded, and instead much of the rhodopsin may still be present in a form that is resistant to solubility. The data also suggest that western blot analysis may not be sufficient to detect proteins that are prone to aggregate, and other methods that do not require protein solubilization should be used.

### Mutant rhodopsin forces the loss of immunoreactivity and mislocalization of wild-type rhodopsin

**Figure 6:**
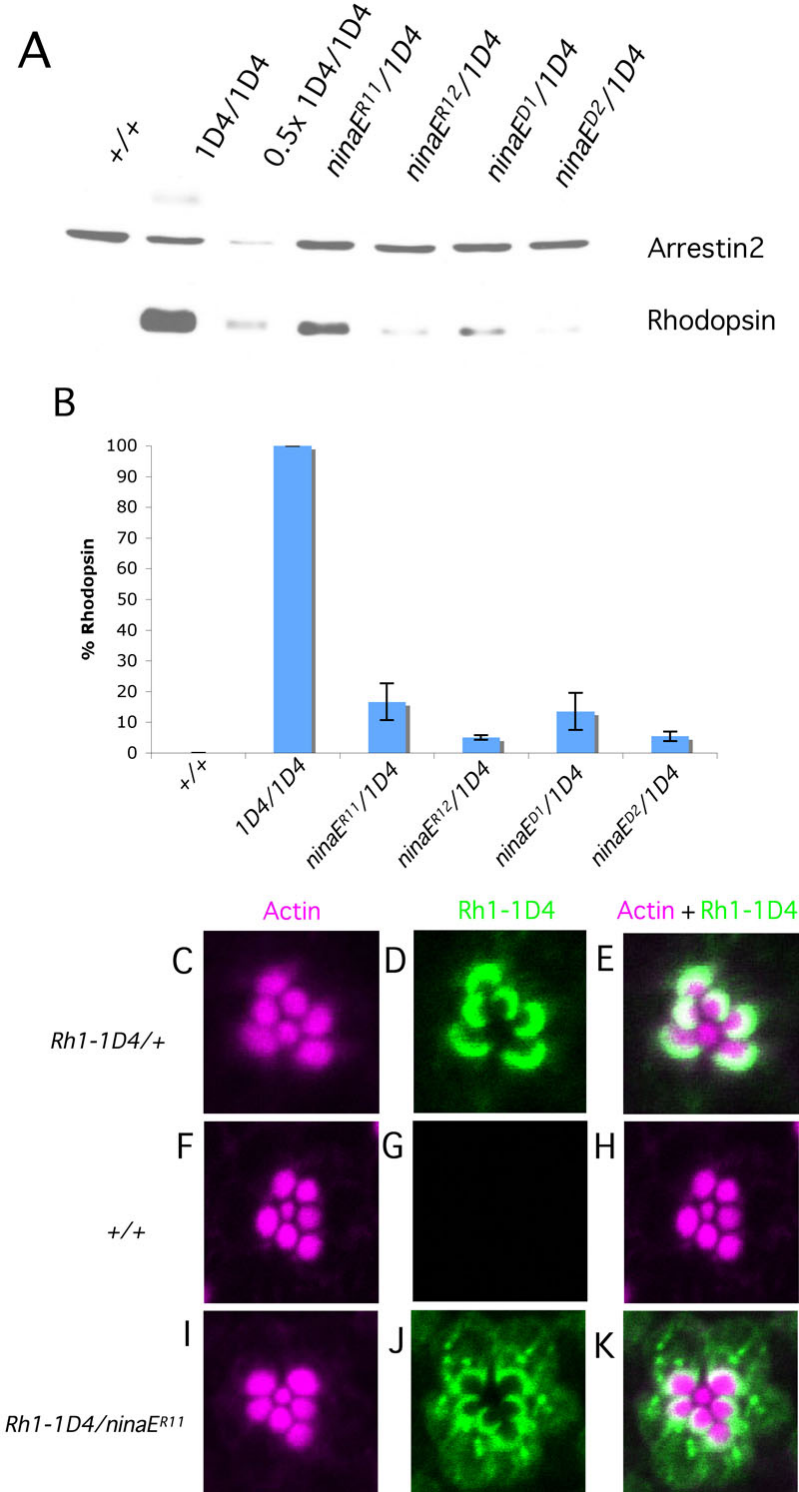
Wild-type rhodopsin quantification and localization in heterozygous *ninaE* mutants expressing rhodopsin with a 1D4 monoclonal antibody tag (Rh1-ID4). **A**: Lysates equivalent of one head were loaded onto each lane and probed with anti-Rh1–1D4 antibody that specifically recognizes wild-type protein. Arrestin2 was used as the loading control. **B**: The blot probed with anti-Rh1–1D4 antibody was quantified to show levels of rhodopsin in mutants relative to the wild type. The reduction in levels of soluble wild-type rhodopsin in heterozygous mutant flies is significant compared to controls (p<0.0001). **C**-**E**: Dark-reared fly retinas were isolated and stained for F-actin and Rh1–1D4. Wild-type photoreceptors show proper rhodopsin localization to the base of the rhabdomeres. **F**-**H**: Flies expressing non-1D4-tagged rhodopsin (+/+) show no staining for rhodopsin, highlighting the specificity of the antibody. **I**-**K**: Flies haploid for mutant *ninaE^R11^* and wild-type Rh1–1D4 show partial proper rhodopsin localization with significant rhodopsin-positive puncta mislocalized to the cell body. Error bars are represented as average % rhodopsin±SD. Each column represents the average of three biologic replicates. Scale bar represents 5 μm.

Our biochemical data indicates that there is a loss of both wild-type and mutant rhodopsin in the heterozygous flies. To establish that mutant rhodopsin expression specifically induces loss of wild-type rhodopsin immunoreactivity, we expressed epitope-tagged rhodopsin in the presence of the rhodopsin variant. We took advantage of a variant of Rh1 where the C-terminus of *Drosophila* Rh1 is replaced by the bovine rhodopsin C-terminus [46]. A monoclonal antibody, ID4, specifically recognizes the C-terminus of bovine rhodopsin [47] and therefore will recognize tagged rhodopsin (1D4) but not endogenous rhodopsin (+/+; Figure 6A, lane 1; Figure 6F-H). In this way we can monitor the presence of wild-type rhodopsin independently from mutant rhodopsin.

When ID4-tagged wild-type rhodopsin (Rh1–1D4) is expressed along with mutant rhodopsin, there is a significant decrease in immunoreactivity of tagged wild-type rhodopsin. The decrease in immunoreactivity on immunoblots closely parallels the data observed when all Rh1 is analyzed (Figure 6A). Compared to flies expressing Rh1–1D4 alone, we detected 16.6% rhodopsin in flies of *ninaE^R11^/Rh1–1D4* (p<0.01), 5% in *ninaE^R12^/Rh1–1D4* (p<0.0001), 13.5% in *ninaE^D1^/Rh1–1D4* (p<0.01), and 5.4% in *ninaE^D2^/Rh1–1D4* (p<0.0001; Figure 6B). This loss of wild-type rhodopsin indicates that mutant rhodopsin is specifically triggering the loss of immunoreactivity of wild-type rhodopsin in this assay.

Since we have demonstrated that mutant rhodopsin is forcing the loss of solubility, which is likely due to misfolding of wild-type rhodopsin, we predicted that wild-type rhodopsin is also mislocalized in the mutant photoreceptors. We chose to look at *ninaE^R11/+^* flies specifically as we had observed multiple Rh1-positive puncta in the cell body of dark-reared animals. Immunolocalization data demonstrate that there are significantly more wild type Rh1–1D4-positive vesicles mislocalized to the cell body compared to haploid Rh1–1D4 flies alone (Figure 6C-E, I-K). We were unable to perform slot blots using the 1D4 antibody, as is shown in Figure 5. However, even though there are severely reduced levels of Rh1 on western blots for *ninaE^R11^/Rh1–1D4* animals, there are detectable levels using immunofluorescence, indicating that Rh1 is still present but is in a conformation that cannot be revealed using western analysis.

Overall, our data indicate that mutant rhodopsin expression exerts its dominant effect by inducing mislocalization and insolubility of wild-type rhodopsin. This results in the likely impaired clearance and accumulation of rhodopsin that may underlie the cause of cell death.
